# Hail‐induced mortality of Asian Openbill (*Anastomus oscitans*) in Southern Tropical China

**DOI:** 10.1002/ece3.8983

**Published:** 2022-06-11

**Authors:** Mingxia Zhang, Lin Wang, Jiabin Li, Qiaoyan Wang, Aidong Luo

**Affiliations:** ^1^ Key Laboratory of Ecology of Rare and Endangered Species and Environmental Protection (Guangxi Normal University) Ministry of Education Guilin China; ^2^ 12388 Guangxi Key Laboratory of Rare and Endangered Animal Ecology Guangxi Normal University Guilin China; ^3^ Southeast Asia Biodiversity Research Institute Chinese Academy of Sciences & Center for Integrative Conservation Xishuangbanna Tropical Botanical Garden Chinese Academy of Sciences Mengla China; ^4^ Xishuangbanna National Nature Reserve Jinghong China

**Keywords:** bird, exotic species, extreme climate event, Xishuangbanna

## Abstract

Hailstorms has been reported to cause mortality of mammals or birds around the world, but the effect of hailstorms on tropical avian species has seldomly been documented. In April 2020, a hailstorm hit Xishuangbanna in south China and was reported to kill 45 Asian Openbills. We estimated the effect of hail by doing fieldwork and interviews. We walked along transects to survey the local avian diversity 3 days after the hail; checked the dead species along the transect; and also interviewed 67 local villagers in 14 villages in the impacted area. We found no evidence that other species were killed by the hail and recorded 40 bird species along the transects in April. Four months later, we surveyed the same transects and recorded 38 species, and the Asian Openbill stayed as one of the most dominant bird species. We concluded that the Asian Openbill is more vulnerable to hail compared with other local birds, but this single hail event did not have an obvious long‐term impact on the population. The result provided an important case study for a tropical bird's response to extreme climate events and we suggested more similar observations to be made in the future.

## Graphical abstract

We reported the death of 45 Asian Openbill (*Anastomus oscitans*) in hail in the tropical area of Southern China, this is the first report of hail‐caused bird mortality in the region. We found the Asian Openbill is the only species killed by the hail, but no obvious fluctuation was observed for the species’ population four months later. This research provides important information for bird's response to climate change.

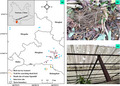

## 1 INTRODUCTION

Understanding how climate change affects diversity distribution is crucial for environment management (Liu et al., 2020). Besides the gradual change of the climate such as mean‐temperature, precipitation, sea‐level etc., an Extreme Climatic Event (ECE) can also affect the survival rate of birds, extirpate local population of mammal or invertebrate species (Gardner et al., 2017; Maxwell et al., 2019). ECE can be defined from both climatological or biological aspect, and here we defined it as a rare, intense event that happens less than a certain percent (e.g., 10%, 5%) of the time in the history of a region's climate (van de Pol et al., 2017). ECE are expected to become more frequent during climate change and potentially cause high mortality (Wingfield et al., 2017). Observation of a single ECE cannot be statistically representative of the phenomenon, while as a case study it still provides opportunities to understand the response mechanism of different species to extreme conditions (Altwegg et al., 2017).

Hail has been reported to cause mammal or bird mortality in North America and Canada (Diehl et al., 2014; Hall & Harvey, 2007; Smith & Webster, 1955). Narwade et al. (2014) reported that more than 62,000 birds of 35 species were killed by several hailstorms in Maharashtra, India, indicating that the hailstorm could severely damage tropical wildlife. Here, we report a severe hail event from April 2020 in Xishuangbanna, tropical area of south China and explore its’ effect on local avian communities.

The Asian Openbill (*Anastomus oscitans*) is a widely distributed species in South and South‐east Asia (Elliott et al., 2020). It was first recorded in China in Dali, Yunnan province, in 2006 (Jiang & Ning, 2010). It then steadily spread across most of the Yunnan province and southern China, and by 2015 a flock of 1100 was reported from the area (Han et al., 2016; Liu et al., 2015). The species occurs in wetland areas such as (fish) ponds, lakes, rivers, ephemeral puddles, or swamps, but also in rice paddies or perched in woodland (Li et al., 2013; Liu et al., 2015; Yang et al., 2019). In our study site, Xishuangbanna, the Asian Openbill likes to forage in wetlands with low forest coverage and away from human disturbance, and has been observed all year round since 2012. Flock sizes here range from 4 to 100, but no breeding populations were detected in the whole Xishuangbanna district (with a total area around 19,000 km^2^) during our two years continuous bird survey or by other local bird watchers (The China Bird Report, 2022 updated).

In the afternoon of the 24th of April 2020, a hailstorm hit Menghun and Mengzhe towns in Menghai county of Xishuangbanna, the hailstorm started around 2:00 pm and lasted for 15–30 min, producing hailstones estimated at 1–5 cm diameter based on video and damage holes (personal observation). The hailstorm damaged large area of tea plantation and rice paddies, as well as some of local residents’ solar water heaters and roof (https://www.chinanews.com.cn/sh/shipin/cns‐d/2019/04‐24/news813053.shtml), and has been reported to kill 45 Asian Openbills. We conducted field surveys three days after the hail. We combined these surveys with interview data to explore the composition of the local bird community, and how many bird species were killed by the hail. Then we conducted a field survey in the same transects four months later, to see whether the local population of Asian Openbill or any other relatively common bird species declined in the aftermath of this event.

## 2 METHODS

### 2.1 Study area

**FIGURE 1:**
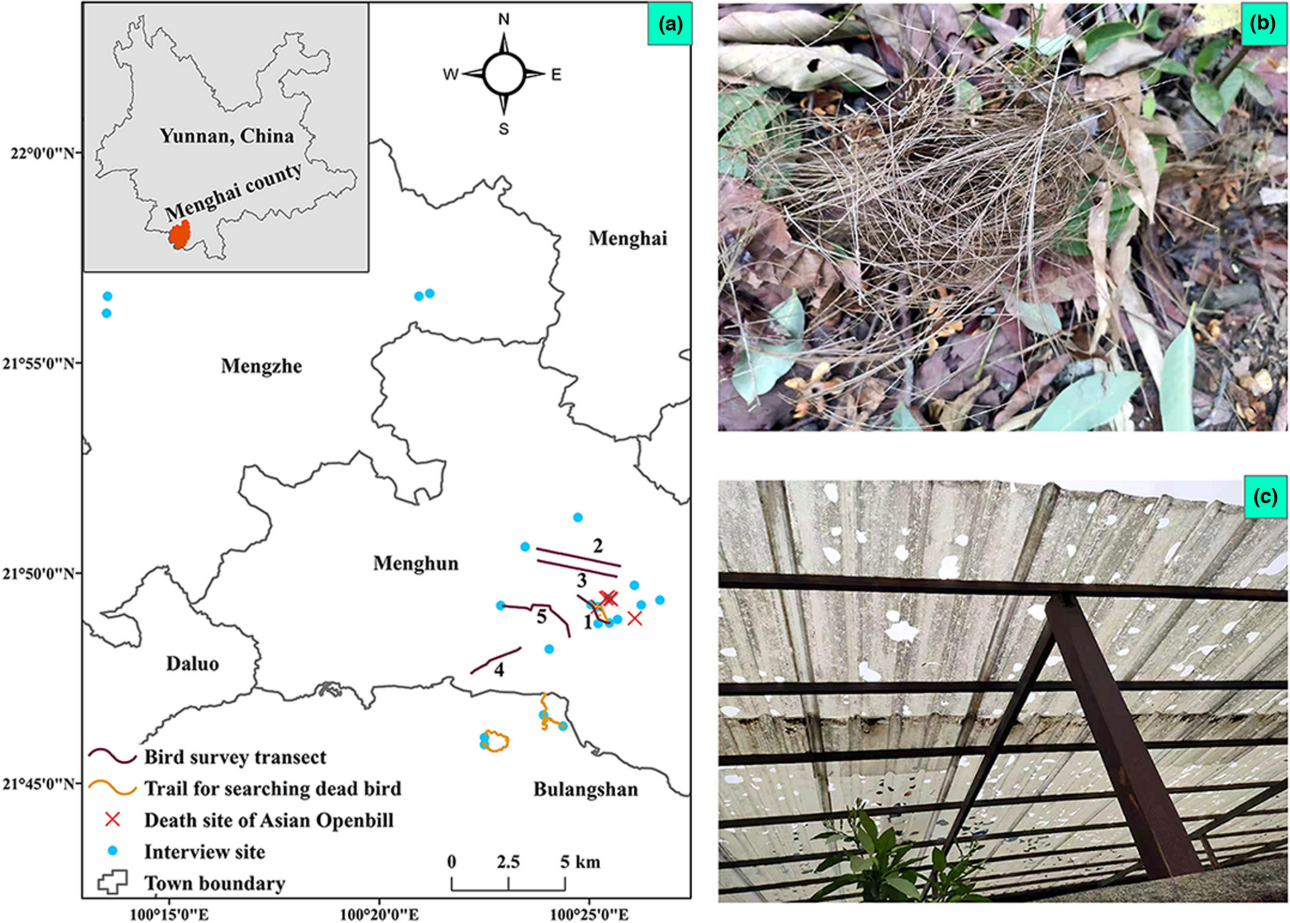
Our study site located in Menghai county of Yunnan province in southwest China, including Menghun, Mengzhe, and Bulangshan towns. The transects for bird surveys are numbered with numbers from 1 to 5 (a). A hail in 24th April 2020 ruined a Munia nest (b) and tea protection shield destroyed by the hail (c)

The study area includes Menghun, Mengzhe towns, and small area of Bulangshan town close by. The towns are in Xishuangbanna, Yunnan province in southwest China, which lies at the northern edge of tropical area (21°45′N to 21°58′N, and 100°12′ to 100°28′E), with a total area of around 200 km^2^ (Figure 1) (Zhu et al., 2006). We checked the hailstorm information online and then drive to the towns to find the most seriously affected villages. We spent three days to locate the seriously affected villages and the places where the Asian Openbill's carcasses were collected. The main land cover types are evergreen forest and agricultural lands, including tea garden and rice paddies, the latter being the dominant type of habitat around our transects. Paddies are typically inundated in April but drained in August, although some small ponds are left for duck raising. The elevation ranges from 1130 to 1790 m, and the southwest monsoon divides the whole year into rain (May to October) and dry (November to April of next year) seasons (Zhang & Cao, 1995). Hail events are quite rare in the study area. We inquired with the local Bureau of Meteorology, but hail records were unavailable. Both the fourth and fifth authors have lived in Xishuangbanna area for more than two decades, and they recalled that hail only occurred around 6–10 times in last decade, and they did not recall a hail event of this magnitude.

### 2.2 Field survey

Transects were set in paddies of the three towns, to get the data of bird diversity and explore the long‐term effect of hail on local bird communities. Five transects were surveyed from 28th April to 2nd May and from 30th July to 5th August (Figure 1a). The first and third authors walked at a velocity of 1 km/h along the trail in the paddies and recorded the distance and angle relative to the transect of all birds seen or heard. The length of each transect was around 2 km, the distance between two transects was no less than 0.5 km, and the surveys were conducted during 8 am to 11 am in the morning, and 4 pm to 7 pm local time in the afternoon.

We searched the paddies of the four villages with the most serious damage in the study area three days after the hailstorm. More than eight km of trails in the paddies were checked for dead birds. The trails were walked at a constant speed, and 50 m at both sides of trails were checked for large bird (>30 cm) and other species' corpses.

### 2.3 Interview

The news of Asian Openbill's death was released online by Menghai forestry bureau (https://www.sohu.com/a/392601566_120672051). We approached the staff we know in this bureau to check the numbers and species of the dead birds. Then structured interviews were conducted in 14 villages within six days (from 26th April, 2020 to 30th April, 2020) after the hail. We used purposive sampling to select respondents who could tell us the most about their experience of the hail event (Bernard, 2017). This was more appropriate than probability sampling because the event was rare and quick, and only certain people experienced it fully enough to provide useful information. First, we drove around the three towns and tried to find out specific villages been hit by the hailstorm, then we walked in the paddies around those villages, and talked with farmers who were working in their cropland. We also went into villages and asked villagers who could tell us about their experience during the hailstorm. Oral consent was obtained for every interviewee, and we told them that they can stop at any time if they feel uncomfortable about the questions. Questions were asked about the damage of the hail, and which species were seen to be killed (Appendix S1 for Questionnaire). We prepared pictures of nine species of local common birds, from which respondents picked out species they had seen killed.

All field surveys were performed in accordance with the relevant Chinese laws and regulations. The interview was approved by the Biomedical Ethics Expert Committee of Xishuangbanna Tropical Botanical Garden, Chinese Academy of Sciences (XTBG‐2021‐06). The informed consent was obtained from all participants.

## 3 RESULTS

According to information online and interview with local bureau staff, they totally received 45 bird carcasses right after the hail on 24th, together with 15 wounded ones. Of the wounded, 10 were released and 3 died on the 25th, while the remaining two were kept in the local rescue center. Among all of the local staff's photos and videos of dead or injured birds that we checked, only Asian Openbills were represented. We surveyed five transects for bird diversity data. In April, 40 species were observed (Table 1). In total, there were four species with abundances larger than 100: Asian Openbill (*Anastomus oscitans*, 294), Eastern Cattle Egret (*Bubulcus ibis*, 234), Scaly Munia (*Lonchura punctulate*, 107), and Red Avadavat (*Amandava amandava*, 105). These four species comprise 75% of the total number. Other species include members of Passeriformes, Pelecaniformes, and Gruiformes. Four months after the hail, these five transects were surveyed again in the study area, and 38 species were observed (Table 1), There were four species with a total abundance larger than 100: Tree Sparrow (*Passer montanus*, 242), Asian Openbill (*Anastomus oscitans*, 303), Eastern Cattle Egret (*Bubulcus ibis*, 337), and Barn Swallow (*Hirundo rustica*, 106). The above four species also comprised 75% of total number. The difference in abundance of Asian Openbill between two surveys is insignificant (Paired *t* test, *t* = −0.0251, *df* = 4, *p*‐value = .98). Totally, we observed 56 species on the five transects.

**TABLE 1:** Bird diversity in study area, three days after hail and 4 months later

English Name	Scientific Name	Residential status^a^	Apr	Transect No. in Apr	Abundance^b^ in Apr	Aug	Transect No, in Aug^c^	Abundance^b^ in Aug
Cattle Egret	*Bubulcus ibis*	R	√	1,3,5	0–205,46.8 ± 88.9	√	1,3,5,2	0–155, 67.4 ± 56.1
Asian Open bill	*Anastomus oscitans*	V	√	1,3,2	0–255,58.8 ± 110.9	√	All	2–230, 60.6 ± 96.0
Scaly Munia	*Lonchura punctulata*	R	√	3,5,2,4	0–45,21.4 ± 20.6	√	All	3–33, 16.8 ± 15.0
Zitting Cisticola	*Cisticola juncidis*	W	√	all	4–35, 17.4 ± 12.4	√	All	4–31, 15.8 ± 11.1
Red Avadavat	*Amandava amandava*	R	√	3,5,2	0–55,21 ± 25.1	0	n	n
Tree Sparrow	*Passer montanus*	R	√	1,3,2,4	0–25, 11.6 ± 9.6	√	1,5,2,4	0–101, 48.4 ± 50.2
Egret	*Egretta garzetta*	R	√	3	0–11, 2.2 ± 4.9	√	1,5	0–5, 1.2 ± 2.2
Ruddy‐breasted Crake	*Zapornia fusca*	R	√	5,2,4	0–9, 2.8 ± 3.7	√	All	1–11, 4.0 ± 4.1
Plain Prinia	*Prinia inornata*	R	√	3,2	0–9, 2.6 ± 4.0	0	n	n
Paddyfield Pipit	*Anthus rufulus*	R	√	1,3	0–3, 1 ± 1.4	√	5,2,4	0–2, 1.0 ± 1.0
Barn Swallow	*Hirundo rustica*	R,P	√	2	0–7, 1.4 ± 3.1	√	All	2–52, 21.2 ± 25.9
Striated Swallow	*Cecropis striolata*	S	√	1	0–5, 1 ± 2.2	√	1,4	0–10, 2.4 ± 4.3
Red‐whiskered Bulbul	*Pycnonotus jocosus*	R	√	4	0–5, 1 ± 2.2	√	4	0–5, 1.0 ± 2.2
Wood Sandpiper	*Tringa glareola*	W	√	3	0–4, 0.8 ± 18	√	3	0–1, 0.2 ± 0.4
Common Tailorbird	*Orthotomus sutorius*	R	√	1,4	0–2, 0.6 ± 0.9	√	4	0–7, 1.4 ± 3.1
Yellow Wagtail	*Motacilla tschutschensis*	P	√	3	0–3, 0.6 ± 1.3	0	n	n
Yellow‐bellied Prinia	*Prinia flaviventris*	R	√	5	0–3, 0.6 ± 1.3	√	4	0–1, 0.2 ± 0.4
Blue‐throated Barbet	*Psilopogon asiaticus*	R	√	4	0–3, 0.6 ± 1.3	√	4	0–4, 0.8 ± 1.8
Hill blue Flycatcher	*Cyornis banyumas*	S	√	4	0–3, 0.6 ± 1.3	0	n	n
Grey‐breasted Prinia	*Prinia hodgsonii*	R	√	1,5	0–1, 0.4 ± 0.5	√	5	0–5, 1.6 ± 2.3
Yellow‐breasted Bunting	*Emberiza aureola*	W	√	3	0–2, 0.4 ± 0.9	0	n	n
Black‐shouldered Kite	*Elanus caeruleus*	S,P	√	3,5	0–1, 0.4 ± 0.5	√	3,5	0–2, 0.6 ± 0.9
Dusky Warbler	*Phylloscopus fuscatus*	W	√	5	0–2, 0.4 ± 0.9	0	n	n
Pin‐striped Tit‐babbler	*Mixornis gularis*	R	√	1	0–1, 0.2 ± 0.4	0	n	n
Glossy Ibis	*Plegadis falcinellus*	V	√	3	0–1, 0.2 ± 0.4	0	n	n
Siberian Blue Robin	*Larvivora cyane*	P	√	3	0–1, 0.2 ± 0.4	0	n	n
Purple Heron	*Ardea purpurea*	R	√	3	0–1, 0.2 ± 0.4	0	n	n
White‐breasted Waterhen	*Amaurornis phoenicurus*	R	√	5	0–1, 0.2 ± 0.4	√	1	0–1, 0.2 ± 0.4
Common Moorhen	*Gallinula chloropus*	R	√	5	0–1, 0.2 ± 0.4	√	3	0–13, 2.6 ± 5.8
Brown Shrike	*Lanius cristatus*	W,P	√	5	0–1, 0.2 ± 0.4	0	n	n
Chestnut‐eared Bunting	*Emberiza fucata*	R,S,W	√	5	0–1, 0.2 ± 0.4	0	n	n
Pied Bush Chat	*Saxicola caprata*	R	√	5	0–1, 0.2 ± 0.4	0	n	n
Great Coucal	*Centropus sinensis*	R	√	2	0–1, 0.2 ± 0.4	√	5	0–2, 0.6 ± 0.9
Siberian Stonechat	*Saxicola maurus*	R,W	√	2	0–1, 0.2 ± 0.4	0	n	n
Asian Barred Owlet	*Glaucidium cuculoides*	R	√	4	0–1, 0.2 ± 0.4	√	4	0–1, 0.2 ± 0.4
Yellow‐bellied Warbler	*Abroscopus superciliaris*	R	√	4	0–1, 0.2 ± 0.4	0	n	n
Oriental Magpie Robin	*Copsychus saularis*	R	√	4	0–1, 0.2 ± 0.4	0	n	n
Indian White‐eye	*Zosterops palpebrosus*	R,W	√	4	0–1, 0.2 ± 0.4	0	n	n
Spotted Dove	*Streptopelia chinensis*	R	√	4	0–1, 0.2 ± 0.4	0	n	n
Sooty‐headed Bulbul	*Pycnonotus aurigaster*	R	√	4	0–1, 0.2 ± 0.4	√	4	0–3, 0.6 ± 1.3
Little Ringed Plover	*Charadrius dubius*	R	0	n	n	√	3,5	0–10, 4.0 ± 5.5
Greater Painted‐snipe	*Rostratula benghalensis*	W	0	n	n	√	3,5,2	0–9, 2.6 ± 3.8
Intermediate Egret	*Ardea intermedia*	R	0	n	n	√	1,3,5	0–9, 2.8 ± 3.7
Cinnamon Bittern	*Ixobrychus cinnamomeus*	S	0	n	n	√	3,5	0–4, 1.2 ± 1.8
Great Egret	*Ardea alba*	S	0	n	n	√	2	0–1, 0.2 ± 0.4
Long‐tailed Shrike	*Lanius schach*	R	0	n	n	√	4	0–2, 0.4 ± 0.9
House Swift	*Apus nipalensis*	S	0	n	n	√	5,4	0–1, 0.4 ± 0.5
White‐throated Kingfisher	*Halcyon smyrnensis*	R	0	n	n	√	4	0–2, 0.4 ± 0.9
Grey Canary Flycatcher	*Culicicapa ceylonensis*	R	0	n	n	√	4	0–2, 0.4 ± 0.9
Slaty‐breasted Rail	*Lewinia striata*	R	0	n	n	√	4	0–1, 0.2 ± 0.4
Red‐wattled Lapwing	*Vanellus indicus*	R	0	n	n	√	5	0–1, 0.2 ± 0.4
David's Fulvetta	*Alcippe morrisonia*	R	0	n	n	√	4	0–1, 0.2 ± 0.4
Common Sandpiper	*Actitis hypoleucos*	W,P	0	n	n	√	4	0–1, 0.2 ± 0.4
Bersra	*Accipiter virgatus*	R	0	n	n	√	4	0–1, 0.2 ± 0.4
White Wagtail	*Motacilla alba*	R,S,W,P	0	n	n	√	2	0–1, 0.2 ± 0.4
Grey‐headed Lapwing	*Vanellus cinereus*	W	0	n	n	√	2	0–1, 0.2 ± 0.4

^a^ R: resident; S: summer visitor; W: winter visitor; P: passenger (Zheng, 2021).

^b^ The abundance is shown in min–max, mean ± standard deviation.

^c^ n means no record in the transect.

In addition to the bird transects, eight km of trails were checked for dead birds three days after the hail event (Figure 1). We found one munia (*Lonchura* spp.) nest that fell from the branch, but no dead birds were detected (Figure 1b).

Sixty‐seven farmers from 14 villages were interviewed about the damage of hail. The age of respondents varied from 24 to 65. Seventy‐two percent were men and 28% were women. Thirty‐eight (55.9%) of them could identify the most common species and said that they had seen dead Asian Openbill. These respondents reported no other species killed. Nineteen Asian Openbills carcasses (30% of the birds sent to local authority) were collected by respondents of our surveys from four different places in the paddy and sent to the local forestry and grassland administration, indicating that our sample was representative. Four of them showed us videos of dead Asian Openbill on Wechat or Douying (popular social media Apps in China), 15 interviewees mentioned that their tea sheds were destroyed by the hailstorm, one said that the hailstorm damaged the tea sheds of all (130) the households in their village, and one villager said the solar water heater of her family has been broken by the hail stones.

## 4 DISCUSSION

From the field observations and the interviews, we found that the evidence of immediate casualties was most evident in Asian Openbills relative to co‐occurring bird species. Although the intensity of the hail is small comparing with other similar studies (Fiss et al., 2019; Hall & Harvey, 2007; Narwade et al., 2014), our single observation provides an important case study for the response of tropical bird species to hail storm. Hailstorms are known to damage agriculture crops, cause loss and poverty to farmers (Battaglia et al., 2019; Jindal, 2015), and promote plant invasion (Peltzer & Wilson, 2006). In light of expected increases in extreme climatic event frequency in the future (Raupach et al., 2021), we suggest more similar observations to be made to monitor severe weather impacts on local bird communities.

Our field surveys three days after the hailstorm showed that the Asian Openbill remained one of the most abundant birds in the area, and while no obvious population fluctuation was observed four months later, this may be due to the small geographic range and short duration of hail. Narwade et al. (2014) found many more dead birds in tropical India, this may due to the higher frequency, larger affected area of hailstorms, and higher bird density in their study area. We acknowledge that our comparison of April and August surveys is only appropriate for resident species that do not naturally vary seasonally in their abundance and detectability. The August populations of locally breeding species might be lower than expected, provided that breeding should be expected to add more individuals to the population. So the lack in difference between April and August counts could be indicative of a negative population impact when put into this context. Our survey was also limited due to lack of comparative data from adjacent, unaffected areas. In addition, we found one destroyed munia nest, indicating our survey might have ignored some small forest birds such as munia or sunbird etc. The munia is brown and small thus it is difficult to detect a munia carcass on forest ground. Moreover, the carcass of birds could be scavenged by domestic dogs/cats or local small carnivores such as Leopard Cat (*Prionailurus bengalensis*), Yellow‐throated Marten (*Martes flavigula*) and rats, and some dead birds could be decomposed quickly, considering the high decomposition rate in tropical zone (Chin et al., 2008). More information should be collected for forest species if similar events happen in the future, and new technology such as weather radar can be used to detect such damage (Carver et al., 2017).

We found two roosting sites of the local water birds during the survey and observed that the Asian Openbill likes to occupy the crown of the roost trees while other birds stay at lower stratus; however, this may not explain Asian Openbill's increased mortality, because the hailstorm occurred during the daytime when all of the water birds were foraging in the paddy field, and all the dead Asian Openbills were found in the paddy. We did not find any evidence of mortality such as freshly scattered feathers or bones around the roosting trees. Together with the Asian Openbill (body size 66–81 cm), we documented Cattle Egret (body size 46–53 cm) (https://birdsoftheworld.org/bow/home) in the paddy, but none of the Cattle Egret (*Bubulcus ibis*) were recorded to be killed, indicating Cattle Egret might either have stronger body structure or different fleeing behavior away from the hail. Badenhorst (1956) found that 500 European Storks (*Ciconia ciconia*) were killed in a hailstorm in South Africa, and many of the storks got wounds on the wings; Wuczyński (2020) reported the death of a Lesser Whitethroat (*Sylvia curruca*) in hailstorm due to fright molt (the ejection of rectrices in fright). We did not get the opportunity to inspect the carcass of Asian Openbills, but no loss of rectrices was observed from the video or pictures, so the Asian Openbills could be killed due to physical wounds caused by the hailstones in this case.

Lei and Liu (2021) found the Asian Openbill population in China is not self‐sustained and the local conditions meet the “tolerance niche” (Sax et al., 2013) for the species. We did not record the age classes of the population in August, but we did not observe any nests or hatchlings during our survey. We suggest continued long‐term observation of the local avian community to see whether the Asian Openbill breed in the study area or not, and explore the ecological response of bird diversity to future similar changes.

## AUTHOR CONTRIBUTIONS

**Mingxia Zhang:** Conceptualization (lead); Funding acquisition (lead); Investigation (supporting); Writing – original draft (lead); Writing – review & editing (equal). **Lin Wang:** Conceptualization (equal); Funding acquisition (equal); Writing – original draft (equal); Writing – review & editing (equal). **Jiabin Li:** Formal analysis (equal); Investigation (equal); Methodology (equal). **Qiaoyan Wang:** Investigation (equal); Methodology (equal). **Aidong Luo:** Investigation (equal); Methodology (equal).

## CONFLICT OF INTEREST

The authors declare no conflict of interest.

## Supporting information

Appendix S1

## Data Availability

The coordinates of the transects and original data are available at Zhang, Mingxia (2021), Transect survey data of the manuscript, Dryad, Dataset, https://doi.org/10.5061/dryad.p5hqbzkr0.

## References

[ece38983-bib-0001] Altwegg, R. , Visser, V. , Bailey, L. D. , & Erni, B. (2017). Learning from single extreme events. Philosophical Transactions of the Royal Society B: Biological Sciences, 372, 20160141. 10.1098/rstb.2016.0141 PMC5434092 28483871

[ece38983-bib-0002] Badenhorst, M. (1956). The effect of a hailstorm on wintering European storks. Ostrich, 27, 89–90.

[ece38983-bib-0003] Battaglia, M. , Lee, C. , Thomason, W. , Fike, J. , & Sadeghpour, A. (2019). Hail damage impacts on corn productivity: A review. Crop Science, 59, 1–14. 10.2135/cropsci2018.04.0285

[ece38983-bib-0004] Bernard, H. R. (2017). Research methods in anthropology: Qualitative and quantitative approaches. Rowman & Littlefield.

[ece38983-bib-0006] Carver, A. R. , Ross, J. D. , Augustine, D. J. , Skagen, S. K. , Dwyer, A. M. , Tomback, D. F. , & Wunder, M. (2017). Weather radar data correlate to hail‐induced mortality in grassland birds. Remote Sensing in Ecology and Conservation, 3, 90–101. 10.1002/rse2.41

[ece38983-bib-0007] Chin, H. C. , Marwi, M. A. , Salleh, A. F. M. , Jeffery, J. , Kurahashi, H. , & Omar, B. (2008). Study of insect succession and rate of decomposition on a partially burned pig carcass in an oil palm plantation in Malaysia. Tropical Biomedicine, 25, 202–208. 19287358

[ece38983-bib-0009] Diehl, R. H. , Bates, J. M. , Willard, D. E. , & Gnoske, T. P. (2014). Bird mortality during nocturnal migration over Lake Michigan: A case study. The Wilson Journal of Ornithology, 126, 19–29. 10.1676/12-191.1

[ece38983-bib-0010] Elliott, A. , Garcia, E. F. J. , Boesman, P. F. D. , & Kirwan, G. M. (2020). Asian Openbill (*Anastomus oscitans*), version 1.0. In J. del Hoyo , A. Elliott , J. Sargatal , D. A. Christie , & E. de Juana (Eds.), Birds of the world. Cornell Lab of Ornithology.

[ece38983-bib-0011] Fiss, C. J. , McNeil, D. J. , Rodríguez, F. , Rodewald, A. D. , & Larkin, J. L. (2019). Hail‐induced nest failure and adult mortality in a declining ground‐nesting forest songbird. The Wilson Journal of Ornithology, 131, 165–170. 10.1676/18-15

[ece38983-bib-0031] Gardner, J. L. , Rowley, E. , de Rebeira, P. , de Rebeira, A. , & Brouwer, L. (2017). Effects of extreme weather on two sympatric Australian passerine bird species. Philosophical Transactions of the Royal Society B: Biological Sciences, 372, 20160148. 10.1098/rstb.2016.0148 PMC5434098 28483863

[ece38983-bib-0012] Hall, D. W. , & Harvey, T. M. (2007). Mortality at a night roost of Great‐tailed Grackles and European Starlings during a spring hail storm. The Wilson Journal of Ornithology, 119, 309–312. 10.1676/05-150.1

[ece38983-bib-0013] Han, L. , Han, B. , Liang, D. , & Gao, G. (2016). Range expansion of Asian open‐billed storks in Southwest China. Sichuan Journal of Zoology, 35, 149–153.

[ece38983-bib-0014] Jiang, A. , & Ning, Y. (2010). A new distribution site of the Asian open‐billed stork (*Anastomus oscitans*) in southwestern China. Chinese Bird, 1, 259–260. 10.5122/cbirds.2010.0020

[ece38983-bib-0015] Jindal, K. (2015). Impact of changing climate on productivity of apple in Himalayas: Urgent need for mitigation of hail damage. In M. L. Choudhary, V. B. Patel, M. W. Siddiqui, & S. Sheraz Mahdl (Eds.), Climate dynamics in horticultural science (pp. 55). Apple Academic Press.

[ece38983-bib-0016] Lei, Y. , & Liu, Q. (2021). Tolerance niche expansion and potential distribution prediction during Asian openbill bird range expansion. Ecology and Evolution, 11(10), 5562–5574. 10.1002/ece3.7456 34026029 PMC8131807

[ece38983-bib-0017] Li, Z. , Dong, Y. , & Yang, H. (2013). Food habitat selection of *Anastomus oscitans* during migration via Xishuangbanna. Forest Inventory and Planning, 38, 25–28.

[ece38983-bib-0018] Liu, Q. , Buzzard, P. , & Luo, X. (2015). Rapid range expansion of Asian Openbill *Anastomus oscitans* in China. Forktail, 31, 118–120.

[ece38983-bib-0032] Liu, X. , Rohr, J. R. , Li, X. , Deng, T. , Li, W. , & Li, Y. (2020). Climate extremes, variability and trade shape biogeographical patterns of alien species. Current Zoology, 67, 393–402. 35386252 10.1093/cz/zoaa068 PMC8979237

[ece38983-bib-0033] Maxwell, S. L. , Butt, N. , Maron, C. A. , McAlpine, S. , Chapman, A. , Ullmann, D. , Segan, B. , & Watson, J. E. (2019). Conservation implications of ecological responses to extreme weather and climate events. Diversity and Distributions, 25, 613–625.

[ece38983-bib-0019] Narwade, S. , Gaikwad, M. C. , Fartade, K. , Pawar, S. , Sawdekar, M. , & Ingale, P. (2014). Mass mortality of wildlife due to hailstorms in Maharashtra, India. Bird Populations, 13, 28–35.

[ece38983-bib-0020] Peltzer, D. A. , & Wilson, S. D. (2006). Hailstorm damage promotes aspen invasion into grassland. Botany‐Botanique, 84, 1142–1147.

[ece38983-bib-0021] Raupach, T. H. , Martius, O. , Allen, J. T. , Kunz, M. , Lasher‐Trapp, S. , Mohr, S. , Rasmussen, K. L. , Trapp, R. J. , & Zhang, Q. (2021). The effects of climate change on hailstorms. Nature Reviews Earth Environment, 2, 213–226. 10.1038/s43017-020-00133-9

[ece38983-bib-0022] Sax, D. F. , Early, R. , & Bellemare, J. (2013). Niche syndromes, species extinction risks, and management under climate change. Trends in Ecology & Evolution, 28, 517–523. 10.1016/j.tree.2013.05.010 23769712

[ece38983-bib-0023] Smith, A. G. , & Webster, H. R. (1955). Effects of hail storms on waterfowl populations in Alberta, Canada: 1953. The Journal of Wildlife Management, 19, 368–374. 10.2307/3797388

[ece38983-bib-0008] The China Bird Report . (2022). http://www.birdreport.cn/

[ece38983-bib-0024] Van de Pol, M. , Jenouvrier, S. , Cornelissen, J. H. , & Visser, M. E. (2017). Behavioural, ecological and evolutionary responses to extreme climatic events: Challenges and directions. Philosophical Transactions of the Royal Society B: Biological Sciences, 372, 20160134. 10.1098/rstb.2016.0134 PMC5434086 28483865

[ece38983-bib-0025] Wingfield, J. C. , Pérez, J. H. , Krause, J. S. , Word, K. R. , González‐Gómez, P. L. , Lisovski, S. , & Chmura, H. E. (2017). How birds cope physiologically and behaviourally with extreme climatic events. Philosophical Transactions of the Royal Society B: Biological Sciences, 372, 20160140. 10.1098/rstb.2016.0140 PMC5434091 28483870

[ece38983-bib-0034] Wuczyński, A. (2020). Hail‐induced nest mortality and possible fright molting of a passerine bird during the pre‐incubation period. The Wilson Journal of Ornithology, 132, 476–481.

[ece38983-bib-0026] Yang, J. , Yang, X. , Lei, Y. , & Liu, Q. (2019). Nocturnal roost use and roosting tree selection of Asian openbill (*Anastomus oscitans*) in Mengzi, Yunnan. Acta Ecologica Sinica, 39, 5371–5377.

[ece38983-bib-0027] Zhang, J. , & Cao, M. (1995). Tropical forest vegetation of Xishuangbanna, SW China and its secondary changes, with special reference to some problems in local nature conservation. Biological Conservation, 73, 229–238. 10.1016/0006-3207(94)00118-A

[ece38983-bib-0028] Zheng, G. (2021). A checklist on the classification and distribution of the birds of China (3rd ed.). Chinese Science Publishing & Media Ltd.

[ece38983-bib-0029] Zhu, H. , Cao, M. , & Hu, H. (2006). Geological history, flora, and vegetation of Xishuangbanna, Southern Yunnan, China 1. Biotropica, 38, 310–317.

