# Supplementary material for: Hail‐induced mortality of Asian Openbill (Anastomus oscitans) in Southern Tropical China
Source: Ecol Evol. 2022 Jun 11;12(6):e8983. doi: 10.1002/ece3.8983 (PMC9188030; doi:10.1002/ece3.8983)
Supplement: Supplementary file 1 — Appendix S1 [file ECE3-12-e8983-s001.docx]

**Appendix 1. Questionnaire**

Interviewer: No. of Questionnaire:

Hello, we are from local institute, we want to ask about the effect of the hail to the bird, could you take 15 minutes to answer our questions? We won’t ask your name, and this interview won’t bring any impact to your life.

1. Gender F/M
2. How many years have you lived in this town?
3. 1-5 yrs
4. 6-10 yrs
5. Over 10 yrs
6. How does this hailstorm affect your life?
7. It has big impact to my life
8. It has small impact to my life
9. It has no impact to my life
10. Do you know of any bird have been killed by this hailstorm? （Yes to 5，No to 6）

Yes□ No□

1. Did you see it by yourself or hear from others, or hear the news from social media?

By myself□ Social media such as wechat, douyin□ Hear from my friend□ Other□

If the interviewee saw it from social media, ask him/her to show the interviewer the picture and ask him/her forward the picture to us, and ask about the picture taking place.

If the interviewee saw it by him/herself and took picture, ask him/her forward the picture to us. If there’s no pictures, ask the interviewee to pick up species from the pictures.

If the interviewee only shows us Asian openbill, we will continue to show them the remaining species and ask whether they saw these species killed by the hail. We will record the species, number, and place if the interviewee chooses any species.

| Species* | Number | place | Environment** | Death | wounded | Note*** |
| --- | --- | --- | --- | --- | --- | --- |
|  |  |  |  |  |  |  |
|  |  |  |  |  |  |  |
|  |  |  |  |  |  |  |
|  |  |  |  |  |  |  |

**：A:Road B:Shrub land C:forest D:Field E:Wetland F:River

* : ①Egret ②Pond Heron ③Asian Openbill ④Cattle Egret ⑤Red-whiskered Bulbul ⑥Sooty-headed Bulbul ⑦Great Barbet ⑧Magpie Robin ⑨Crimson Sunbird

***：V:Video P：Picture

Note：

1. Do you remember any hailstorms happening here before? Was any bird killed by those storms?

1. Except the birds, do you know/see any other animal species that was killed by this hailstorm?

| Species | Number | Place | Environment* | Death | wounded | Note** |
| --- | --- | --- | --- | --- | --- | --- |
|  |  |  |  |  |  |  |
|  |  |  |  |  |  |  |
|  |  |  |  |  |  |  |

*：A:Road B:Shrub land C:forest D:Field E:Wetland F:River

**：V:Video P：Picture

1. How old are you？(optional) years
2. What is your job？（optional）
3. I’m a farmer
4. I do business
5. I work in company
6. I work in government

Thanks for your help! This is a small gift to express our gratitude, do you have any question for me?
